# Single-cell and coupled GRN models of cell patterning in the *Arabidopsis thaliana *root stem cell niche

**DOI:** 10.1186/1752-0509-4-134

**Published:** 2010-10-05

**Authors:** Eugenio Azpeitia, Mariana Benítez, Iliusi Vega, Carlos Villarreal, Elena R Alvarez-Buylla

**Affiliations:** 1 Laboratorio de Genética Molecular, Desarrollo y Evolución de Plantas, Instituto de Ecología & Centro de Ciencias de la Complejidad (C3), Universidad Nacional Autónoma de México, Ciudad Universitaria, Coyoacán, México D.F. 04510. México; 2 Instituto de Física, Universidad Nacional Autónoma de México, Ciudad Universitaria, Coyoacán, México D.F. 04510. México Apdo. Postal 20-364. México D.F., 04510, Mexico & Centro de Ciencias de la Complejidad (C3), Universidad Nacional Autónoma de México, Ciudad Universitaria, Coyoacán, México D.F. 04510. México

## Abstract

**Background:**

Recent experimental work has uncovered some of the genetic components required to maintain the *Arabidopsis thaliana *root stem cell niche (SCN) and its structure. Two main pathways are involved. One pathway depends on the genes *SHORTROOT *and *SCARECROW *and the other depends on the *PLETHORA *genes, which have been proposed to constitute the auxin readouts. Recent evidence suggests that a regulatory circuit, composed of *WOX5 *and *CLE40*, also contributes to the SCN maintenance. Yet, we still do not understand how the niche is dynamically maintained and patterned or if the uncovered molecular components are sufficient to recover the observed gene expression configurations that characterize the cell types within the root SCN. Mathematical and computational tools have proven useful in understanding the dynamics of cell differentiation. Hence, to further explore root SCN patterning, we integrated available experimental data into dynamic Gene Regulatory Network (GRN) models and addressed if these are sufficient to attain observed gene expression configurations in the root SCN in a robust and autonomous manner.

**Results:**

We found that an SCN GRN model based only on experimental data did not reproduce the configurations observed within the root SCN. We developed several alternative GRN models that recover these expected stable gene configurations. Such models incorporate a few additional components and interactions in addition to those that have been uncovered. The recovered configurations are stable to perturbations, and the models are able to recover the observed gene expression profiles of almost all the mutants described so far. However, the robustness of the postulated GRNs is not as high as that of other previously studied networks.

**Conclusions:**

These models are the first published approximations for a dynamic mechanism of the *A. thaliana *root SCN cellular pattering. Our model is useful to formally show that the data now available are not sufficient to fully reproduce root SCN organization and genetic profiles. We then highlight some experimental holes that remain to be studied and postulate some novel gene interactions. Finally, we suggest the existence of a generic dynamical motif that can be involved in both plant and animal SCN maintenance.

## Background

Stem cell (SC) research has received much attention during the last decade [1], as these cells are the source of new pluripotent cells in plants and animals and are fundamental for the maintenance of tissues during adulthood. Hence, understanding the dynamics and molecular genetics of SC niches (SCNs) has become a central question in biological and medical research [2,3]. Interestingly, SCNs share important structural and dynamic characteristics across distantly related multicellular organisms [3-6], which suggests the existence of underlying generic mechanisms. Thus, the study of plant SCNs, which are often more amenable to experimental and modeling studies than those of animals, may help researchers understand some such generic traits and may shed light on issues related to human health [7].

In contrast to animals, structures arise throughout the whole life cycle of plants from active SCNs, which are exposed in the so-called meristems. *Arabidopsis thaliana *has two main SCNs. One of these is in the Shoot Apical Meristem (SAM), located at the tip of the aerial part of the plant, and another is located in the Root Apical Meristem (RAM), at the acropetal end of the primary root. The *A. thaliana *root and root SC niche (SCN) are well described at the anatomical level. The root SCN includes four cells that rarely divide and constitute the quiescent center (QC), surrounded by four sets of initial cells that give rise to the different types of differentiated cells in the root (i.e., stele, cortex, endodermis, epidermis, lateral root-cap and columella cells) [8].

Besides the thorough anatomical characterization of this system, some of the molecular components that are necessary to establish and maintain the *A. thaliana *RAM and its SCN cellular patterning have been uncovered and characterized only recently (Table 1). One of these components implicates the module of *SHORTROOT *(*SHR*), its target gene *SCARECROW *(*SCR)*, the immediately downstream genes of the dimer SHR/SCR and other genes that interact with them. Another regulatory circuit includes the *PLETHORA *(*PLT*) genes, which have been proposed to be key components of the molecular readout of the plant hormone auxin [9-12].

**Table 1:** Summary of the experimental evidence

INTERACTIONS	EXPERIMENTAL EVIDENCE	REFERENCE
***SHR →SCR***	The expression of *SCR *is reduced in *shr *mutants. ChIP-QRTPCR experiments show that *SHR *directly binds *in vivo *to the regulatory sequences of *SCR *and positively regulates its transcription.	[9,16]
***SCR →SCR***	In the *scr *mutant background promoter activity of *SCR *is absent in the QC and CEI. A ChIP-PCR assay confirmed that *SCR *directly binds to its own promoter and directs its own expression.	[10,14]
***JKD →SCR***	*SCR *mRNA expression as probed with a reporter lines is lost in the QC and CEI cells in *jkd *mutants from the early heart stage onward.	[17]
***MGP--|SCR***	The double mutant *jkd mgp *rescues the expression of SCR in the QC and CEI, which is lost in the *jkd *single mutant.	[17]
***SHR →MGP***	The expression of *MGP *is severely reduced in the *shr *background. Experimental data using various approaches have suggested that *MGP *is a direct target of *SHR*. This result was later confirmed by ChIP-PCR.	[14,16,17]
***SCR →MGP***	*SCR *directly binds to the *MGP *promoter, and *MGP *expression is reduced in the *scr *mutant background.	[14,17]
***SHR →JKD***	The post-embryonic expression of *JKD *is reduced in *shr *mutant roots.	[17]
***SCR →JKD***	The post-embryonic expression of *JKD *is reduced in *scr *mutant roots.	[17]
***SCR →WOX5***	*WOX5 *is not expressed in *scr *mutants.	[24]
***SHR →WOX5***	*WOX5 *expression is reduced in *shr *mutants.	[24]
***ARF(MP) →WOX5***	*WOX5 *expression is rarely detected in *mp *or *bdl *mutants.	[24]
***ARF→PLT***	*PLT1 *mRNA region of expression is reduced in multiple mutants of *PIN *genes, and it is overexpressed under ectopic auxin addition. *PLT1 *&*2 *mRNAs are absent in the majority of *mp *embryos and even more so in *mp nph4 *double mutant embryos.	[11,12]
***Aux/IAA--|ARF***	Overexpression of Aux/IAA genes represses the expression of DR5 both in the presence and absence of auxin. Domains III & IV of Aux/IAA genes interact with domains III & IV of ARF stabilizing the dimerization that represses ARF transcriptional activity.	[22,23]
***Auxin--| Aux/IAA***	Auxin application destabilizes Aux/IAA proteins. Aux/IAA proteins are targets of ubiquitin-mediated auxin-dependent degradation.	[reviewed in [18]]
***CLE40 --| WOX5***	Wild type root treated with CLE40p show a reduction of *WOX5 *expression, whereas in *cle40 *loss of function plants *WOX5 *is overexpressed.	[25]

**Figure 1:**
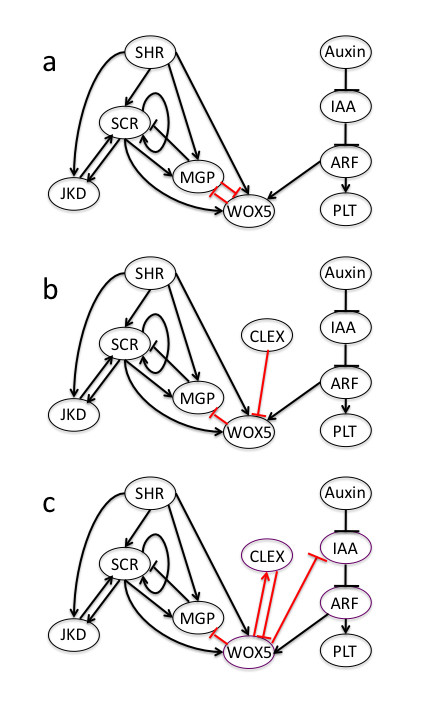
**Root stem cell niche GRN models**. Nodes represent the genes or hormones in the case of auxin. Arrows correspond to activations, and flat arrows correspond to repressions. Four models were tested. Model A and A' (a) differ from B and B' (b) in the *WOX5 *negative regulation. In models A and A', *WOX5 *is downregulated by MGP, whereas in models B and B' *WOX5 *is downregulated by the hypothetical gene *CLEX*. Models A' and B' differ from models A and B in the SCR value in line 14 of their logical rule as shown in Additional file 1. In (c), the GRN used for the coupled GRN model is depicted. Even when *CLE40 *is an experimentally reported data, in our model, we assume negative regulation of *WOX5 *in all SCs, so *CLEX *is treated as a novel prediction. In all GRNs, red arrows indicate the novel postulated interactions, and black arrows indicate interactions based on experimental data. Purple nodes in (c) are the nodes involved in the activator-inhibitor motif. The *IAA *node in the GRN graph represents the *Aux/IAA *gene family, not auxin. Of all the interaction considered here, the dimer SHR/SCR activating *SCR *and *MGP*, auxin repressing Aux/IAA and Aux/IAA repressing ARF had been experimentally confirmed as direct interactions.

Experimental evidence used to generate the four single cell GRNs. These previously reported results were the basis for the interactions postulated in the SCN GRN models graphs of figure 1.

*SHR *is a gene that is expressed in the stele at the transcriptional level; its protein then moves to the adjacent cellular layer (i.e., cells in the QC, endodermis-cortex initials (CEI) and endodermis), where it activates *SCR *[13]. SCR is necessary for its own activation in the QC and CEI [10,14]. Both genes have been implicated in the maintenance of the RAM and SCN and the radial organization of the root [9,10,15]. SHR and SCR interact through their central domains; together, they control the transcription of several genes [14,16]. *MAGPIE *(*MGP*) is a target gene of SHR/SCR and has been implicated in the regulation of the root radial pattern, although its function is not yet fully understood. *JACKDAW (JKD) *is expressed in the QC and CEI. Mutations in *JKD *lack *SCR *expression in the QC and CEI, causing a misspecification of the QC, which is perhaps due to its effect on *SCR *expression. Yeast two-hybrid assays have shown that SHR, SCR, JKD and MGP can physically interact, which suggests that protein-protein complexes among them are involved in SC regulation [17].

In addition to the SHR/SCR SCN regulation, *PLT *genes are also necessary for the maintenance of the root SCN. The double mutant *plt1 plt2 *fail to maintain the SCN, and in this mutant, eventually all cells in the RAM differentiate [11]. *PLT *genes act redundantly, and *plt1 plt2 pl3 *triple mutants are rootless and resemble the Auxin Response Factor (ARF) *monopteros *(*mp*) single mutant. Indeed, *mp *single and *mp arf7/nhp4 (nonphototropic hypocotyl4) *double mutants show reduced or no expression of *PLT1 *and *PLT2 *transcripts from heart stage onward, which suggests that the activation of *PLT *transcription occurs downstream of the ARFs [11,12]. Moreover, application of exogenous auxin increases *PLT *transcription.

The transcriptional activity of the ARFs has been widely studied, and the Aux/IAA proteins have been proposed as their key negative regulators [18-21]. The Aux/IAA proteins repress the transcriptional activity of the ARF forming hetero-dimers. The SCF^TIR1 ^ubiquitin ligase complex promotes Aux/IAA degradation in the presence of auxin [22,23].

Finally, *WUSCHEL RELATED HOMEOBOX 5 *(*WOX5*) is a gene expressed exclusively in the QC. In *wox5 *mutants, the QC fails to maintain correct gene expression and to keep the distal SC undifferentiated. *WOX5 *is hardly detected in *shr*, *scr*, or *mp *[24]. The *WOX5 *distribution is expanded in the *wox5 *background, suggesting that this gene has a negative feedback loop [24]. Recently, *CLAVATA-like 40 *(*CLE40*), a secreted peptide, was found to negatively regulate *WOX5 *expression through *ARABIDOPSIS CRINKLY4 *(*ACR4*) in the more distal part of the meristem [25]. Other studies suggest that additional *CLE-like *genes could be involved in RAM maintenance [26-29].

Despite the thorough description of mutants and paired gene interactions, it still remains unclear how the concerted action of all the studied genes and their regulatory interactions collectively yield the gene profiles (configurations) characteristic of the cell types within the root SCN. Indeed, soon after cells depart from the QC, they attain distinct gene expression configurations, each characterizing a set of SC or initial cell types that eventually give rise to the distinct cell lineages conforming the mature root. How such cellular heterogeneity in SCs is dynamically established while the QC cells are kept undifferentiated, considering that all cells within the SCN have the same genetic information, is still not well understood. Dynamic gene regulatory models are of great value for addressing these issues.

Gene Regulatory Network (GRN) models have proven to be useful tools for studying the concerted action of molecular entities acting during cell differentiation and pattern formation [30-36]. These models are made up of nodes representing genes, proteins or other molecules and edges that stand for the regulatory interactions among these elements [37]. The dynamics of these networks may be described using systems of coupled equations, either continuous or discrete [37,38]. For Gene Regulatory Networks (GRNs) involved in cell fate determination, it has been proposed that their steady-state gene configurations (also referred to as attractors) correspond to gene activation profiles typical of different cell types [39]. Therefore, investigating the dynamics of such GRNs may be key for understanding cell differentiation, cell patterning and morphogenesis during developmental processes.

Some theoretical approaches have addressed lineage specification, regeneration and other aspects of SCNs in animals [40-42] and plants [43,44]. However, to our knowledge, dynamic models that aim at understanding cell-fate determination and patterning in SCNs are still scarce. Specifically, such a model is lacking for the *A. thaliana *root meristem. Hence, although some of the genes necessary for the root SCN specification and maintenance have been identified and functionally characterized [45], there is no dynamic characterization of the whole regulatory module. Additionally, it is still unclear if the molecular components and interactions reported previously are sufficient to dynamically and robustly recover the cell types and patterns of the *A. thaliana *root SCN.

In this paper, we have integrated the available experimental data on root SCN maintenance into discrete GRN dynamic models. We postulate several alternative regulatory modules to investigate if alternative topologies of regulatory interactions, which include those uncovered so far in addition to a few additional predictions, are sufficient to recover genetic profiles characteristic of the main cell types within the SCN.

Given that roots, when exposed to diverse environmental conditions or even to multiple genetic mutations [e.g., [11,17,46-48]], still harbor a normal or almost normal SCN, we hypothesized that the niche cellular patterning should be regulated by a robust GRN. The formal models proposed here enabled tests of such a hypothesis by addressing if the proposed GRN models attained the same gene configurations in the face of transient (e.g., initial conditions or inputs from other modules connected to the one under study) or permanent perturbations. We also investigated the robustness of the models by translating the discrete GRN models to continuous ones and by verifying if the SCN GRN attractors were maintained. Additionally, we validated the proposed GRN models by testing if they also recovered gene configurations of experimentally characterized loss and gain of function mutants. Comparisons of the alternative GRN models tested helped us detect experimental gaps and postulate novel predictions that could guide future experiments.

We also designed a discrete spatial version of coupled GRNs to address if the intracellular GRN coupled by the reported cell-to-cell communication via movement of four of the GRN components could also yield the gene configurations observed in different cell types and positions within the *A. thaliana *root SCN. A local activator and lateral inhibitor motif were included as part of the coupled network model as a prediction, in part given that such a motif has been postulated for the SAM, which has important similarities with the RAM maintenance [49,50].

The results obtained in this work show that the genes that have been characterized in SCN patterning are largely sufficient to recover both the gene configurations observed in the main cell types within the root SCN and the overall spatial pattern of such cells. However, our work strongly suggests that additional components and circuits are still to be discovered, and these may render the root SCN robust in the face of transient perturbations as well as some genetic mutations.

## Results

### Four alternative GRN models sufficient to recover observed gene expression profiles in cells within the root stem cell niche

Based on the experimental data summarized above and in Table 1, we generated a discrete root SCN GRN model (see Methods for details). The regulatory interactions are indicated by arrows (activation) or flat-end edges (repression) in the GRN (Figure 1). It is important to note that even though in figure 1 all the interactions between nodes appear to be direct, the arrows can represent a direct interaction or an interaction mediated by one or more intermediate molecular components (i.e., indirect interaction). We still lack experimental data to discern between these two possibilities in many instances. In figure 1, we indicate which interactions are experimentally confirmed as direct interactions, whereas the rest are indirect. In the logical functions (Additional file 1), *0 *represents a non-functional protein or non-expressed gene, except for *PLT *and auxin, which have a graded expression and for which *0 *represents a level of expression insufficient to exert their function in the SCN.

**Figure 2:**
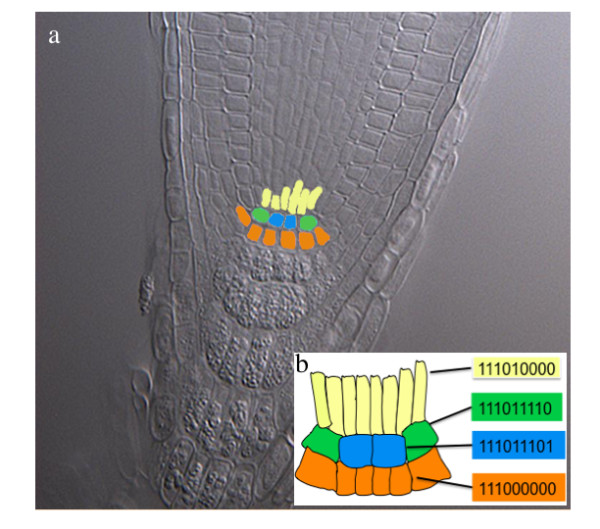
**Simplified cellular pattern of the root stem cell niche compared to a real root**. The four cell types within the SCN, each of which corresponds to one of four stable attractors recovered by the models in figure 1. The vascular initials (yellow), cortex-endodermis initials (green), quiescent center (blue) and columella-epidermis-lateral root cap initials (orange) (b) in the cleared root tip of *Arabidopsis thaliana *colorized to show the corresponding cell types represented by the attractors recovered by the single-cell GRN models in figure 1 and schematized in (a). The activation states of the nodes are represented by *0 *and *1 *in the following order: *PLT*, auxin, *ARF*, *Aux/IAA*, *SHR*, *SCR*, *JKD*, *MGP *and *WOX5*.

A few articles have demonstrated that *SHR *movement depends on both cytoplasmic and nuclear localization [51,52] and its activity depends on its nuclear localization. Our GRN models do not consider how *SHR *or any other node intracellular localization affects in mobility and function. Nonetheless, the logical rules postulated for this gene qualitatively recover and agree with the available data related to both aspects of this gene function. Because each of the *ARF, PLT *and *Aux/IAA *genes have redundant functions and overlapping expression patterns and the particular function of single genes in the SCN is not clear, we collapsed each of these groups of genes into a single node for each gene family (Figure 1). The postulated GRN does not distinguish between columella and epidermis-lateral root cap initials due to lack of experimental evidence, and we thus refer to them as CEpI (for columella epidermis initials) (Figure 2). Hence, we expected only four GRN attractors; namely, those corresponding to the QC, vascular initials, Cortex-Endodermis initials (CEI) and CEpI.

While we were integrating available published data into a preliminary GRN model, we detected experimental gaps or ambiguities in five of the genes considered in the network. All of the gaps and ambiguities concern gene transcriptional regulation and were found in *SHR*, *SCR*, *JKD*, *MGP *and *WOX5 *genes. As far as we know, some of the regulators of these genes have not been discovered or published yet.

Much research has been conducted regarding the function of *SHR*, but we did not find any reported gene directly regulating its transcription. In our model, this result implies that because *SHR *activity does not have any positive or negative input regulator, its final state will depend only on its initial state.

It has also been reported that *scr *and *shr *single mutants severely reduce postembryonic *JKD *expression [17] and that the dimer SHR/SCR positively regulates *MGP *expression [14]. This result means that SCR and SHR are both necessary for a postembryonic wild type expression of *JKD *and *MGP*. Nevertheless, *MGP *expression is absent in the QC, where both *SCR *and *SHR *genes are expressed. Similarly, *JKD *express in a different region than the SHR and SCR region. So, SHR and SCR are not sufficient to explain the *JKD *and *MGP *expression because the region of expression of the latter genes is different than that of *SCR *and *SHR*. Hence, it is likely that *JKD *and *MGP *have additional regulators. However, in the model, we assumed that the latter two genes are only under *SCR *and *SHR *regulation. As mentioned above, also the positive regulators described for *WOX5 *(i.e., *SHR*, *SCR *and *ARF*) are present in the CEI, where *WOX5 *is not expressed, which suggests that there are also uncovered *WOX5 *regulators.

Sabatini and collaborators [10] reported that *SCR *SCN expression depends on itself, but other reports [e.g., [53]] showed that ectopic *SHR *expression alone (i.e., without *SCR *ectopic expression) is able to induce *SCR *expression outside the QC, CEI or endodermis cells. So, it is not well understood why even when SHR protein is present in the vascular initials, *SCR *is not, but when *SHR *is ectopically expressed in epidermis and lateral root cap initials, *SCR *turns on. One possibility is that *SHR *alone is capable of activating *SCR*, but our analysis shows that even if this is the case additional *SCR *regulators are still waiting to be discovered (see details below). This and further analysis demonstrate that even though a great amount of research has been carried out on *SCR *regulation, it is not yet fully clear how this gene expression pattern is maintained in the root SCN. With this in mind, we propose a *SCR *logical rule (see Additional file 1) that together with the logical rules of the genes included in the GRN seems to be sufficient for recovering observed gene expression profiles.

We also noted that given the conditions considered in the GRN, *PLT*, *ARF*, *Aux/IAA *and auxin form a linear pathway with no inputs from other nodes included in the GRN. These nodes always have the same state in the models and contribute little to our understanding of the SCN GRN as far as it has been uncovered, but we decided to keep these nodes for two reasons: i) this pathway has been shown to be important in SCN patterning experimentally, and ii) by including them, we provide a more comprehensive formal framework that may later enable connections to other regulatory modules, such as those controlling the cell cycle or a more realistic auxin transport model, in both of which *PLT *and auxin are known to play essential roles.

The SCN GRN that only incorporated published data was not sufficient to recover the observed gene configurations in the SCN. This first GRN model lead to stable gene configurations that did not include the attractors corresponding to that observed in the QC and the CEI cells, and it also yields an attractor, which combined QC/CEI gene activities. The latter combination has not been observed in any of the wild type *A. thaliana *root SCN cell types. Therefore, we decided to postulate two predictions concerning additional regulatory interactions that, in the context of the root SCN GRN uncovered up to now, are sufficient to recover the expected attractors or stable gene expression configurations that have been described for the different cell-types in the root SCN (Figure 2 and table 2 and 3). Our simulations showed that by assuming a down-regulation of *WOX5 *in the CEI and of *MGP *in the QC, the modeled GRN models were sufficient to recover the observed gene expression configurations.

**Table 2:** Simulated configurations of models A and A' compared to those observed in real roots of *Arabidopsis thaliana*

Cell type	PLT	Auxin	ARF	Aux/IAA	SHR	SCR	JKD	MGP	WOX5
**QC**	1(1)	1(1)	1(1)	0(0)	1(1)	1(1)	1(1)	0(0)	1(1)
**Vascular Initials**	1(1)	1(1)	1(1)	0(0)	1(1)	0(0)	0(0)	0(0)	0(0)
**CEI**	1(1)	1(1)	1(1)	0(0)	1(1)	1(1)	1(1)	1(1)	0(0)
**CepI**	1(1)	1(1)	1(1)	0(0)	0(0)	0(0)	0(0)	0(0)	0(0)

A value of *1 *means that the gene is "ON", whereas a value of *0 *means that it is "OFF". Simulated gene states for each cell type are shown first and observed gene states are in parenthesis.

**Table 3:** Simulated configurations of models B and B' compared to those observed in real roots of *Arabidopsis thaliana*

Cell type	PLT	Auxin	ARF	Aux/IAA	SHR	SCR	JKD	MGP	WOX5	CLEX
**QC**	1(1)	1(1)	1(1)	0(0)	1(1)	1(1)	1(1)	0(0)	1(1)	0(0)
**Vascular Initials**	1(1)	1(1)	1(1)	0(0)	1(1)	0(0)	0(0)	0(0)	0(0)	1(1 or 0)
**CEI**	1(1)	1(1)	1(1)	0(0)	1(1)	1(1)	1(1)	1(1)	0(0)	1(1)
**CepI**	1(1)	1(1)	1(1)	0(0)	0(0)	0(0)	0(0)	0(0)	0(0)	0(0)

A value of *1 *means that the gene is "ON", whereas a value of *0 *means that it is "OFF". Simulated gene states for each cell type are shown first and observed gene states are in parenthesis. It is important to note that the extra attractors of the simulated configurations in these models are the same as the one corresponding to the vascular initials but with *CLEX *OFF.

The assumed down-regulations discussed above lack experimental support and hence constitute novel predictions. However, there is, in principle, more than one minimal way to model *WOX5 *inhibition in a manner that is consistent with the rest of the available data and the observed gene configurations within the SCN. In a first model (A), we assumed that *MGP *represses *WOX5 *and *vice versa*. We made this assumption for several reasons: i) the reported conditions for *WOX5 *transcription (i.e., *ARF*, *SHR *and *SCR *expression that positively regulate *WOX5 *expression) are also present in the CEI, and ii) the reported conditions for *MGP *transcription are observed in the QC (i.e., *SHR *and *SCR *expression that positively regulate *MGP *expression). *WOX5 *and *MGP *expression patterns are complementary, so even when we are aware that *MGP *and *WOX5 *possibly do not regulate each other directly, our assumption considers a potential indirect regulation.

In another model (B), we assumed that the proximal expression of *WOX5 *is negatively regulated by an unknown gene that could be a *CLE-like *gene. We decided to use the *CLEX *name for this hypothetical regulator of *WOX5 *because recent evidence demonstrated that *CLE40 *inhibits *WOX5 *expression [25]. We did not use *CLE40 *directly because the hypothetical regulator (*CLEX*) should have a region of expression or activity wider to that reported for *CLE40*. Also, *CLEX *could represent more than one gene, including *CLE40. *Moreover, it is important to also acknowledge that the role of the node marked in our GRN model by *CLEX *could, in fact, involve other genes as well. For example, Williams and collaborators [54] proposed that HD-ZIPIII genes regulate *WUS *expression in the SAM. Several similarities between the SAM and RAM SCNs have been described [e.g., [24]]. In the SAM, HD-ZIPIII genes are negatively regulated by miRNA165/6, which, in turn, are direct targets of the dimer SHR/SCR [55]. HD-ZIPIII genes function is not clear in the root, so including them would not be justified based on the available experimental information. Nonetheless, the *CLEX *could represent the latter or other yet to be uncovered genes. So in model B, the negative regulation of *WOX5 *over *MGP *was kept, but we removed the regulation of *MGP *over *WOX5*. Both A and B models have two distinct versions that differ only in line 14 of the *SCR *logical rule. The A' and B' models have a different assumption in this logical rule than the A and B models (Additional file1). We performed analyses on all four of these models.

To identify the attractors of each model, we used the program *Atalia *[[56]; freely available] by following the dynamics of all possible initial configurations of gene expression. Both versions of model A converge to only four attractors that coincide with experimentally reported gene profiles for the cells within the root SCN (Table 2); namely, QC, CEpI, CEI, and the vascular initials (Figure 2). Both versions of model B converge to five attractors. These attractors correspond to the same four attractors as those recovered with models A and A', but the vascular initials are duplicated with the hypothetical gene *CLEX *being either "ON" or "OFF" in each case (Table 3). This first result already suggests that the proposed GRN models assuming *WOX5 *down-regulation suffices for recovering the expected attractors and, therefore, constitutes a useful tool for exploring the qualitative dynamic traits of the system under study.

We found that in spite of the intricacy and complexity of the regulatory system, the root SCN GRN implies relatively straightforward dynamics, where the activation states of *SHR *and *SCR *determine the final attractor. The lack of *SHR *activity in the GRN unequivocally leads to the CEpI attractor, whereas the presence of *SHR *activity leads to the vascular initials if *SCR *is "OFF" and to the CEI or QC attractor if *SCR *is "ON". This result is confirmed by checking the basins of attraction, where half of the configurations lead to the CEpI attractor, as expected from the dynamics, and the other half lead to vascular initials, QC or CEI depending on the initial *SCR *state. As proposed before [11,12], *PLT *does not seem to be important for cell-fate determination within the SCN but rather for the apical-basal patterning of cell behavior as a read-out of auxin gradients along the longitudinal root axis (Additional file 2). Our models are useful for showing that the two modules important for the SCN patterning (the SHR/SCR and the auxin-PLT) are only connected by *WOX5 *and together render stable gene expression configurations similar to those observed in the main cell types of the root SCN.

### Validation of the single-cell GRN models with simulations for loss and gain of function mutants

To challenge and thus validate the proposed models, we simulated experimentally described mutations and addressed if the recovered gene expression configurations in the simulated mutants corresponded to those observed in the actual root SCN of such lines or could help to pose novel predictions. Gain-of-function mutations were simulated by fixing the over-expressed gene's value to *1 *while fixing the mutated gene's value to *0 *simulated loss-of-function mutants. Most simulated mutants of all models reproduced the gene configurations that have been reported experimentally (Table 4 and 5), but some discrepancies were encountered.

**Table 4:** Simulations of loss of function mutants

Gene	Model A	Model A'	Model B	Model B'	Model A-I
**SHR**	YES	YES	YES	YES	YES
**SCR**	YES	YES	YES	YES	YES
**MGP**	NR	NR	YES	YES	YES
**JKD**	NR	YES	NR	YES	YES
**WOX5**	NC	NC	YES	YES	YES
**PLT**	YES	YES	YES	YES	YES
**ARF**	YES	YES	YES	YES	YES
**Aux/IAA**	YES	YES	YES	YES	YES
**Auxin**	YES	YES	YES	YES	YES

As observed, in most cases simulations recovered the experimentally observed configurations. When we did not recover the expected genetic configurations, we distinguish two situations: not recovered (NR), which indicates that even though experimental data was available it was not recapitulated and not enough data for comparison (NC), which indicates that experimental data was lacking, so in these cases a comparison could not be done. The coupled GRN results are reported in (A-I).

**Table 5:** Simulations of gain of function mutants

Gene	Model A	Model A'	Model B	Model B'	Model A-I
**SHR**	NR	NR	NR	NR	YES
**SCR**	NC	NC	NC	NC	NC
**MGP**	NC	NC	NC	NC	NC
**JKD**	NC	NC	NC	NC	NC
**WOX5**	YES	YES	YES	YES	YES
**PLT**	YES	YES	YES	YES	YES
**ARF**	NC	NC	NC	NC	NC
**Aux/IAA**	YES	YES	YES	YES	YES
**Auxin**	NC	NC	NC	NC	NC

When we did not recover the expected genetic configurations, we distinguish two situations: not recovered (NR), which indicates that even though experimental data was available it was not recapitulated and not enough data for comparison (NC), which indicates that experimental data was lacking, so in these cases a comparison could not be done. The coupled GRN results are reported in (A-I).

Welch and collaborators [17] reported that in *jkd *loss-of-function mutants, *SCR *transcription in the SCN diminished or disappeared and also showed miss-specified QC cells, but the CEI were not lost. In concordance with this, the *SCR *logical rules of our A and B models determine that *SCR *expression is lost if *JKD *is not present. When we simulate *jkd *loss of function, *WOX5*, which marks our QC attractor, is still expressed, and *SCR *does not disappear in the QC, but it does cause the loss of the CEI attractor. These results contradict the observed gene profile pattern of *jkd*. We reasoned that because experimental *jkd *mutants still have CEI and keep *QC25 *expression until 8-9dpg [17], which is SCR-dependent, JKD function could be enhancing *SCR *transcription to a wild-type level; however, in a *jkd *background, SCR could remain expressed and functional at a low level. This hypothesis implies that JKD is dispensable for *SCR *expression or SCR function. We simulated the latter possibility by altering line 14 of the *SCR *truth table (this change produced our A' and B' versions of models A and B, respectively), which allowed *SCR *transcription in *jkd*. In fact, by making this change, we could recover the *jkd *loss of function phenotype (i.e., we did not lose CEI as observed in this mutant), and we predicted that even when the QC is miss-specified, *WOX*5 may remain active, at least for as long as *QC25 *remains active. In this case, *SCR *must also remain expressed, but at a lower level than in wild type. These simulated alterations of the truth tables suggest a need for further experiments (see discussion).

*mgp *loss-of-function single mutant does not have a visible experimental phenotype, but in model A and A' the CEI attractor disappears, and the initial conditions that originally lead to this attractor now lead to the QC attractor. Models B and B' do not show any altered profiles when a loss-of-function *mgp *is simulated. This result coincides with what is observed experimentally, and such a result depends upon the introduction of the hypothetical gene *CLEX *into the models. Our simulations predict that *CLEX *over-expression suffices for the consumption of the QC.

It is well documented that *PLT *genes are key regulators of SC identity and maintenance [11,12], but their direct target genes have not been found. When we mutated the *PLT *node, in the four models, the only effect observed was a lack of expression or constitutive expression of this component, which depended on whether or not we were simulating a loss or gain-of-function mutation, respectively. To further verify the validity of our model and gain insights about the role of *PLT *activity in the root SCN GRN, we added a *PINFORMED *gene (namely *PINX*) and *QC46, *a QC marker to our GRN models, both of which have been experimentally found to be under the control of *PLT *and other genes already considered in our GRN models [11,46]. We decided to use the generic name *PINX *and not a specific *PIN *because it has been reported that *PLT *genes regulate the expression of more than one *PIN *gene, and several *PIN *genes are expressed in the root SCN. By including these genes, we recovered the genetic configuration observed in *PLT *loss-of-function mutant, which also lacks *PINX *and *QC46*, thereby verifying that an adequate activity of *PLT *was being simulated in our models. *PINX *and *QC46 *were introduced in the GRN models only for this analysis.

We were unable to fully validate other gain of function simulations because data on the additional markers for columella and epidermis markers, as well as crosses of over-expression lines with cell-marker lines, are lacking (Table 5). The only two discrepancies found between our simulations and observed configurations concerns *jkd *in models A and B and *mgp *in models A and A'. These discrepancies lead to novel predictions (see discussion section and Table 4).

In conclusion, all of our analyses suggest that the regulatory module proposed here in various versions is indeed largely sufficient for explaining most of the cell-fate determination gene expression profiles in the SCN. The latter is true for the wild type and most mutant cases reported up to now. Our simulations suggest that model B', which assumes that *CLEX *is a negative regulator of *WOX5 *and that *SCR *expression is independent of JKD activity, renders gene expression configurations reproducing the available experimental data. However, it is intriguing that model B' is not as robust to perturbations as models A and A' (see below). This lack of robustness could be due to the introduction of the *CLEX *node, which is necessary to repress *WOX5 *activity without a *MGP *loss of function phenotype but may interact with *WOX5 *in a way that is different to that assumed here. Nonetheless, these analyses illustrate that dynamic GRN models, like the ones used here, are useful tools to test how single gene mutations may yield contrasting stable gene configurations depending on the overall network topologies. It is interesting to note that configurations and cellular patterns may be drastically affected by some relatively small changes in the logical rules of certain genes but are not affected by a great majority of alterations.

### The recovered cell-type gene configurations are robust to genetic perturbations

The above analyses already show that the recovered gene configurations are robust to transient gene modifications because all possible initial configurations lead to a few attractors, which overall correspond to configurations observed in the different types of cells within the root SCN. However, to test the robustness of the uncovered SCN GRN module to genetic alterations, we performed simulations to explore alterations in which node's logical rules yield the greatest modifications in the attractors. To this end, we altered, one by one, the output of every logical rule and ran the system to recover all the attractors from all the possible initial configurations of each altered network. We found that for B and B' and for A and A', 55.4% and 62.85%, respectively, of the tested alterations do not yield novel attractors or cause any of the originally encountered ones to disappear. The remaining 44.6% and 37.15% of the alterations rendered fewer or additional attractors for models A and A' and for B and B', respectively. These results suggest that the postulated SCN GRN models are relatively robust. Nonetheless, other previously characterized GRN for *A. thaliana *cell differentiation have been shown to be more robust than the models proposed here [e.g., [32,33]]. Hence, as an additional robustness test, we decided to perform two additional analyses: i) a Derrida analysis [57-59] to test if the GRN models postulated here are under chaotic, ordered or critical dynamics and ii) a continuous approximation of the Boolean model to address if the same attractors are recovered when the kinetic functions are continuous.

**Figure 3:**
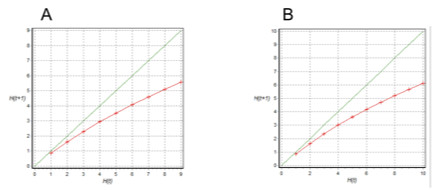
**The Derrida curve of models A and B**. The Derrida test allows for assessment of whether or not the GRN in question is in a chaotic, ordered or intermediate (critical) state. It has been suggested that living systems are located in a critical state, in which they exhibit both a degree of organization and also of flexibility [59]. This analysis is based on a comparison of the trajectories of similar initial conditions. If they diverge rapidly, then the system is said to be chaotic, whereas if they do not diverge or diverge very slowly, the system is said to be ordered. In this graph, it is shown that the curve describing the dynamic of the GRN is very similar to the identity line at the beginning (i.e., for small *t *values) and then diverges, which seems to characterize systems that are in a critical regime [[59] and references there in]. The (A) Derrida curve of model A and (B) Derrida curve of model B. Similar curves were found for model A' and model B'.

The Derrida analysis for the four GRN models in this study was performed using *Atalia*, and we found that the SCN GRN models also exhibit a critical dynamics in the face of perturbations (Figure 3).

Finally, we put forward a continuous version of the discrete GRN model to address if a system of differential equations was able to recover the same attractors. This approach enables us to test if the postulated logical discrete rules imposed artifacts in recovering some of the stable gene configurations, and if using continuous kinetic functions different or additional attractors are recovered. To obtain the system of differential equations, we transformed each discrete function into a differential equation (see methods and Additional file 3 for details). Interestingly, for all of the models postulated and tested in the discrete case, the corresponding continuous models recovered the same attractors, plus an additional unstable attractor in the cases of models A and A'. This extra attractor seems to stem from the assumption that *MGP *acts as a negative regulator of *WOX5*. This extra steady state is between those corresponding to the CEI and QC attractors, with an activation level of 0.5 for both *MGP *and *WOX5*. To calculate the stability of the extra attractor, we ran the dynamics of the continuous system 1000 times, but considering perturbed steady states with alterations of up to 30% of recovered values as the initial conditions. As a result, we found that the extra attractor is rather unstable because it converges to either CEI or QC stable configurations in the face of very small perturbations (see methods for details).

Taken together, the fact that all possible initial configurations only converge to the expected attractors, the analyses done by directly perturbing the logical functions, the continuous approximation, and the Derrida graph analyses confirm that the GRN models studied here are relatively robust. In any case, the fact that these GRN models are not as robust as other GRN models previously studied [e.g., [32,33]] and that the actual root SCN has been shown to be robust to several perturbations [e.g., [11,12,46,48]] suggests that additional redundant circuits, as found in other systems [60], underlie SCN patterning. Additionally, further components of the SCN GRN may still remain undiscovered.

### A model of coupled GRN recovers observed spatial configurations in the root stem cell niche

Recent experimental evidence suggests that *CLE40 *and *WOX5 *behave in a similar way to *WUS *and *CLV3 *in the SAM [24,25], where the latter exerts a lateral inhibition of the former. To simulate such negative regulation in a non-cell autonomous way and to create a model that recovers the spatial cellular configuration observed in the root SCN, we developed a spatial model of coupled single-cell GRNs [e.g., [61]]. We use model B', which, as mentioned before, we believe is the model that best fits the available experimental data.

**Figure 4:**
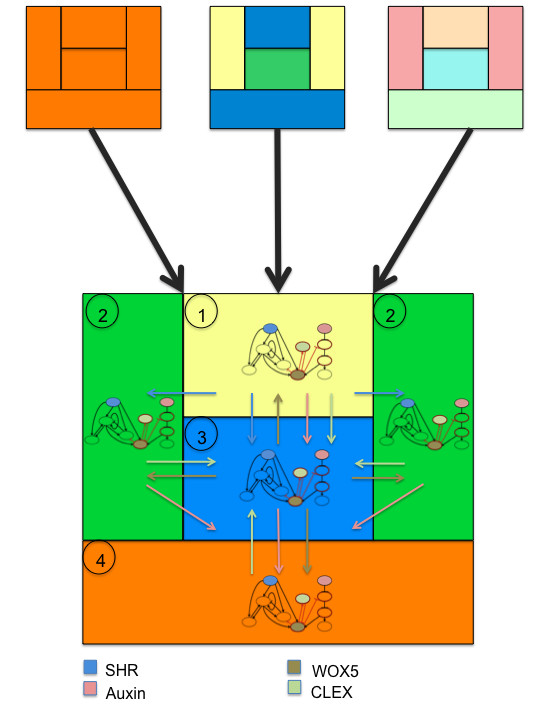
**The coupled GRN**. The four cell types considered in the coupled GRN are shown numbered in circles. They were represented by a GRN one for each attractor recovered in the single-cell models. Color filled nodes are the diffusible or mobile elements of the GRN. Note that these mobile elements can act non-cell autonomously or move among cells. For clarity we only show the main movement directions with arrows with the same color code as that used for the network nodes which movement is allowed. All the movements allowed in the model are listed in additional file 4. The figures show how, regardless of the initial configuration, the model always converges to the same attractor with the same spatial structure.

We simplified the cellular structure of the root SCN by considering four types of cells, one for each attractor found in the previous single cell GRN models, arranged symmetrically based on their observed spatial location (Figure 4). Such an arrangement recovers the main qualitative aspects of the SCN cellular pattern. The spatial information in this coupled GRN model was incorporated by considering cell-to-cell movement or the non-cell autonomous action of four of the intracellular components, namely *SHR*, *WOX5*, *CLEX *and auxin, according to experimental data. Based on each cell's spatial position, only certain directions of movement or communication between cells were allowed according to published data. The mobility patterns were fixed during the GRN dynamics.

*CLEX *and *WOX5 *in one cell can affect the logical rules of all other cells (simulating *CLEX *diffusion and non-cell autonomous action of *WOX5*), whereas *SHR *and auxin were only able to affect the rules of certain SCN cells, according to experimental evidence (simulating acropetal active transport in the case of auxin and the role of SCR in constraining SHR movement). Hence, *CLEX *and *WOX5 *activity in one cell affect all neighboring cells. SHR is assumed to move from any cell where it is expressed to any other cell, but its movement is only allowed if *SCR *is not expressed in the same cell, as previously reported [9,13,14].

In the model, auxin moves acropetally according to published data, which demonstrated that this hormone is transported by the *PIN *efflux facilitators to the SCN through vascular, endodermis and cortex cells [46]. It is known that, from the columella initials, auxin can move in many directions, but because we did not consider cells below the columella initials, these auxin movements were not included in the model.

The spatial information provided to the cells by the four mobile network components was incorporated into the logical rules of each network component, yielding a model of 40 different components. These 40 components correspond to ten nodes per intracellular network multiplied by four types of cells, which are distinguished by the mobile elements. The latter affect the logical rules of each one of the components, depending on the spatial position of each cell in which they are found with respect to other cells (positional information) within the niche, as explained above. Hence, in the new meta-GRN model, each component is identified by its node's identity (i.e., the gene or molecule that it represents) and the spatial location where it is found, which is distinguished by the initial letter of the attractor expected there (Figure 4; and see Methods for further detail).

The updating dynamics of the intracellular GRN and of the intercellular movement of the mobile components were assumed to be synchronous and acted in a short-range. The latter is justified by experimental data [62]. The logical rules used for the meta-GRN are found in Additional file 4.

Some assumptions were made because of a lack of data or for simplicity. First, because auxin comes from the upper cells, which are not considered in our model, we fixed the auxin value to *1 *in the vascular initials and CEI where it can move to the QC and then into CEpI as mentioned above. Also, given the recent evidence on the similarities between the SAM and root SCN, we assumed an activator-inhibitor motif for the RAM SCN consisting of *WOX5 *local auto-regulation enhancing the auxin signaling pathway. The latter could be due to a repression of *Aux/IAA *genes, which is suggested by the fact that *WUS *represses type *A-ARR *genes in the SAM thus enhancing ARF transcription activity. This could also be achieved if WOX5 up-regulates *ARF *transcription directly or through auxin synthesis, as has been suggested for auxin homeostasis before [63]. We tested all alternative coupling patterns, and all of them yielded the same result; therefore, we kept the negative regulation of *Aux/IAA *genes by *WOX5*. In [63], it was also reported that auxin addition up-regulates *WOX5 *expression, so in such a circuit, the *ARF *node positively regulates *WOX5 *[24,63], and *WOX5 *positively regulates *CLEX*, which is assumed to inhibit *WOX5 *[25]. Given that *WOX5 *is exclusively expressed in the QC and that *CLE40 *and other *CLE *genes are found outside the QC [64], we assumed that *WOX5 *non-cell autonomously activates *CLEX *outside the QC, but not within the QC. All of these assumptions regarding *WOX5 *and *CLEX *interactions and functions give rise to an activator-inhibitor motif in our root SCN GRN. It is important to note that the activator-inhibitor motif can explain many observed and suggested behaviors of the root SCN, such as the robustness (see discussion).

Finally, it is well known that *SHR *is exclusively expressed in the vascular cells [9], but no transcriptional regulators have been uncovered for this gene. In the vascular cells, SHR does not activate *SCR *[9], but is able to move into the QC, CEI and endodermis cells [13], where it activates *SCR *expression [9]. Based on these, *SHR *output was fixed to *1 *in the vascular initials, and *SCR *transcription was not allowed there in the model. The impossibility to activate *SCR *in the vascular initials was the only topological change made in the meta-GRN model with respect to the single-cell GRN model B'.

We found that to recover the observed gene expression configurations in the right cell and spatial location, it was necessary to set *SCR *to *1 *in the initial condition. Afterwards, from time t+2 and until the end of the simulation, *SCR *followed its original rule postulated for the single-cell model. This assumption implies that *SCR *basal expression, which cannot be explicitly considered in a Boolean model, is sufficient to activate *SCR *when SHR is present in the CEI and QC. Alternatively, it is possible that *SCR *expression depends on an unknown factor that could be expressed during early embryo development. Later on, *SCR *positive feedback may be required to maintain its own expression (see discussion). Another possibility is that the proposed single-cell GNR architecture, once coupled and solved in the spatial model, is not able to fully recover the observed spatial arrangement of gene configurations because of artifacts derived from the dynamics of the discrete model. To test this latter possibility, we ran the spatial network using our continuous approach (see Methods) and also set the expression state of *SCR *to "ON" in the initial conditions. Notably, with the continuous system we only recovered one attractor in which the observed gene expression configurations found in each cell location mimicked those observed in the root SCN; thus, we recovered the same results as with the discrete version (see below) if we set *SCR *"ON" at the beginning of the simulation. This result supports our prediction that an early acting factor or *SCR *basal expression is necessary for the up-regulation of *SCR *during embryo development.

In the discrete meta-GRN spatial model, we exhaustively explored all possible initial configurations (i.e., 2^40 ^initials configurations) and recovered again the four gene expression configurations that characterize the cell types distinguished in the modeled SCN with only one cellular arrangement that resembles the arrangement found in real roots (Figure 4). This single attractor is attained regardless of the initial configuration used. In figure 4, we exemplify three of the 2^40 ^different initial configurations that converged to the observed one.

We ran the discrete meta-model of four coupled GRNs using the model checker program *ANTELOPE *[Argil J, Azpeitia E, Benitez M, Carrillo M, Rosenblueth D and Alvarez-Buylla E, unpublished data, available upon request]. Model checkers have been widely used for hardware verification, which allow the verification of the different properties in discrete systems. These computational tools are based on a logical analysis and allow verification of different properties, such as the attractors of the system in question. Questions in *ANTELOPE *can be posed by using Hybrid Computational-Tree Logic language, which can be used to verify the properties of any discrete system. The *ANTELOPE *software and a better description of *ANTELOPE *are available upon request. Several accounts on model checking software and Hybrid Computational - Tree Logic are available [65-67].

To validate the spatial model, we simulated mutants that have been documented experimentally. In most cases we recovered the observed gene configurations for each cell type organized in the expected spatial positions (Table 4 and 5). For example, the simulated *SHR *gain of function simulation was able to not only recover the expected mutant configurations but also replaced the CEpI attractor with two different attractors, one corresponding to the CEI attractor and another in which the only difference from the CEpI attractor was the ectopic expression of *SHR. *Such configurations and spatial arrangements coincide with those observed experimentally in this mutant's epidermis-lateral root cap and collumela initials, respectively.

Another example of the mutants analysis corresponds to the *scr *loss-of-function simulation in which the CEI and the QC configurations are lost and the SHR anomaly diffuses to the CEpI, as has been observed experimentally. Simulations of *mgp *do not yield any altered configuration, as has also been reported experimentally. All other simulated mutants recovered configurations that mimic those observed in their corresponding actual mutant plants. In a few cases, simulation results could not be compared to actual mutant configurations because such lines have not been reported yet (Table 5). Such simulations thus constitute novel predictions.

## Discussion

### GRN dynamic models that are sufficient to recover *A. thaliana *root SCN cell gene expression configurations

We have postulated novel, alternative GRN models that constitute the first dynamic regulatory system sufficient to explain how the *A. thaliana *root SCN is maintained. Such models are also able to reproduce cell-type determination and spatial patterning in the root SCN. This result suggests that some key components have been uncovered and that these components, given some additional newly predicted interactions, are sufficient to recover gene expression configurations that resemble those known for the main cell types within the root SCN. This study adds to previous ones that have shown the utility of using qualitative models to understand cell differentiation and spatial cellular patterning during development of other systems [30-33,35,36].

From the beginning of our analysis, we noted that the recovered GRN models describe a very simple dynamics, which are congruent with previous intuitive or schematic static models [e.g., [11]]. Still, several characteristics of the root SCN GRN could not have been predicted or analyzed without a dynamic framework like the one provided here.

For example, schematic models proposed from information available until now about root SCN maintenance have considered two critical modules for *A. thaliana *root SCN establishment and maintenance: i) the *SHR*/*SCR *and ii) the *PLT *pathways involved in the radial cell patterning and the apical-basal gradient of cell behavior. It has been suggested that the intersection or combination of the *PLT *and the *SHR*/*SCR *pathways is both necessary and sufficient for the localization, maintenance and patterning of the root SCN [11]. However, we found that the integration of these two modules into a single GRN dynamic did not explain how the symmetry is broken in the root SCN and how cell pattering is maintained.

As observed in the SCN GRN model based only on experimental evidence, the combination of these two modules did not allow us to reproduce the configurations matching those observed within different cell types in the SCN. Hence, our model shows that the connection of these two pathways via *WOX5 *and the addition of the new element (*CLEX*) are necessary to explain the root SCN cellular pattern observed.

Therefore, we propose that the GRN underlying *A. thaliana *root SCN establishment and maintenance is more complex than previously suggested [11]. To break the symmetry of the apical root meristem, the combination of a radial and an apical-basal circuit are required. Nevertheless, the additional circuits proposed here, which have also been found in other SCN [e.g., [44,49]], are indeed necessary. The missing components could also add robustness to the GRN and are key for establishing and maintaining the cellular heterogeneity observed in the root SCN.

The fact that the results recovered for such a qualitative model are robust to alterations in the logical functions in over 60% of the cases suggests that knowledge regarding the detailed functioning of the genes is not relevant in determining the steady-state gene configurations. Rather, it is the overall topology of the GRN that determines its dynamics and recovered attractors. For example, several details of gene's functions that have been experimentally documented were not included explicitly. For example, this is the case with the movement and function of SHR, which depends on its intracellular localization [51,52]. Nonetheless, the expected role and behavior for this protein were recovered in the proposed models. Additionally, the robustness observed in the *A. thaliana *SCN is not as high as that documented for other small GRNs, which suggests the existence of additional components and/or redundant circuits as have been found in other systems [60,68].

As mentioned above, the analyses of the GRN models proposed here show that even though important components of the GRN underlying the *A. thaliana *root SCN patterning are already known, some are still missing. The existence of some of these gaps was already well known, such as those associated with *SHR *transcriptional regulation, but others were uncovered thanks to the dynamic approach presented here. This approach enabled us to compare simulated gene expression configurations when using GRNs that differed from those reported before.

The fact that all possible configurations attained with the GRNs proposed here converge to only those observed confirms that the SCN GRN is strongly canalized, and that regardless of the initial states used, the systems proposed lead to the expected stable configurations. This feature is also found in the spatial model of coupled GRNs. The strong canalization of these GRN models suggests that they must also be robust. This robustness has been observed experimentally in several studies [e.g., [11,12,46,48]], and has been observed in other previously studied developmental GRNs [e.g., [32,33,69,70]].

Previously studied biological GRNs appear to be near criticality (Figure 3) [58,59] for other biological GRNs as well. Indeed, biological GRNs are expected to be robust in the face of perturbations, but these systems should be also able to respond and adapt to transient and permanent perturbations and thereby exhibit evolvability. Shmulevich and Kauffman [58] predicted that biological GRNs should be on the border between order and chaos, where robustness and evolvability coexist. Balleza and collaborators [59] show that experimentally grounded biological GRNs for bacteria, yeast, *Drosophila *and *A. thaliana *are in fact in the so-called critical state. Such analyses rely on the so-called Derrida analysis [57,59]. We performed this analysis, and strikingly, even when our GRN models show certain degree of robustness to perturbations and a critical dynamics, they are less robust than other GRNs [e.g. [32,33]]. The latter suggests that additional components or redundant circuits that render a higher robustness to alterations are likely to be discovered for the SCN GRN.

Additional robustness in the GRN can come from at least four sources. i) The fact that the *PLT*, *ARF *and *Aux/IAA *nodes actually represent several genes. If these were explicitly modeled, the GRN could become more robust. ii) A cross-talk with other developmental regulatory modules, as recently described [55,71-73], could also confer additional robustness to the SCN GRN. We could not include this cross-talk because important experimental information is still lacking. iii) Additional components that confer dynamic redundancy, and thus additional robustness, to the system could also be missing [60,68]. iv) Finally, additional undiscovered components that, even if they do not confer dynamic redundancy, may increase the GRN robustness.

The four possibilities have been documented in other experimental systems. For example, the root auxin gradient is a robust process, which is redundantly generated by the concerted action of several PIN genes and by the high self-regulating dynamics (composed of many feed forward and feedback loops), which regulates auxin transport, biosynthesis and signaling [18,46,48].

The conversion of the Boolean approach into a continuous one provides the possibility of exploring a richer dynamics of the GRN due to the continuous character of the variables and parameters of the system. It may lead, for example, to a different set of attraction basins. However, the sigmoidal structure of the activation functions involved in the continuous approach implies that the qualitative behavior of the solutions of the differential equation system have only a weak dependence on the specific values of the parameters [74]. In particular, in the limiting case where the activation functions acquire a step-like behavior, we recover the same set of (stable) attractors as those arising from the discrete model and an extra unstable one, as an analysis based on Lyapunov coefficients reveals. Thus, this kind of analysis constitutes an additional robustness test of the system. Furthermore, the continuous approach may become useful for future more sophisticated developments considering larger spatio-temporal implementations, which take into account cell cycle and signal transduction elements.

### Experimental gaps and predictions

The models developed here are useful to postulate new predictions concerning the GRN underlying the root SCN cellular patterning and to uncover experimental gaps.

The *mgp *loss of function simulation suggested that additional components controlling *WOX5 *expression in the proximal meristem have not yet been found. Specifically, we predict that *WOX5 *is down-regulated by a gene that is able to move to the proximal SCN cells or a gene that is expressed in those cells. We think that this gene (or these genes) could be from the *CLE-like *gene family but are different from *CLE40*. This hypothesis is consistent with published data because several *CLE *genes are expressed throughout the root tissues, including the proximal meristem [29,64] and because even when recent evidence demonstrates that *CLE40 *down-regulates *WOX5 *in the initial cells, *CLE40 *seems to be insufficient for the negative regulation of *WOX5 *in the proximal meristem given that neither *CLE40 *nor *ACR4*, the latter of which perceive CLE40, are expressed there [25].

Another important prediction was derived from comparisons of the A and B vs. A' and B' models, along with simulations to recover *jkd *loss of function and the spatial model analysis. Our analyses suggest that *JKD *could only enhance *SCR *expression rather than being an obligate activator; however, once *SCR *is activated, its activity depends upon its own positive feedback and SHR activity. To verify this hypothesis experimentally, one could assess if a reporter gene under the *SCR *promoter is enhanced when crossed to a 35S:SCR line and if the reporter level of expression is the same or lower in a *jkd *compared to a wild type background.

The latter prediction was complemented by another prediction detected from the spatial model analysis, which dealt with *SCR *transcriptional regulation. The positive feedback loops, like the one sustained by *SCR*, are well studied. They are commonly found as a motif that can provide an efficient switching mechanism, hysteresis, bi-stability and robustness in the presence of noise and is also functional to change response time [75,76]. Hence, in the SCN GRN, the *SCR *positive feedback may give rise to a hysteretic, robust and efficient switching behavior in the face of transient and sometimes permanent perturbations but probably does not regulate the initial expression of *SCR*.

Simulations for *clex *loss-of-function as well as for *SHR*, *SCR*, *MGP*, *JKD *and *WOX5 *gain-of-function lines showed that additional research is needed. For example, data for the lateral root cap-epidermis and collumela initials are scarce [e.g., [77-79]] and simulations concerning them are hard to validate.

Another way to validate the models presented here is to explore their behavior under contrasting environmental or hormonal conditions. To that end, we have also tested the GRN models under different auxin concentrations simulated in discrete steps. We found that the postulated GRN models are able to respond to changes in auxin concentrations in ways that resemble those observed experimentally [80] because the gene configurations recovered in the simulations are similar to those observed in real roots treated with different concentrations of auxin. Detailed results of these simulations are provided in Additional file 2.

### Is there a generic motif for plant and animal SCN patterning?

To explore novel hypotheses concerning cellular patterns in the root SCN, we performed simulations of the coupled GRN. We achieved this by incorporating experimental evidence concerning the cell-to-cell movement of some of the GRN components into the logical rules that govern the dynamics of each cell GRN. Interestingly, such a simple spatial model converged to only one global attractor, which contained the cell-specific stable gene configurations that have been observed in each of the relative spatial locations within the real root SCN. Most importantly, this model was successfully validated, as it was able to recover altered configurations observed experimentally in the corresponding simulated mutants and yielded the same results in the continuous version. In the spatial model, we incorporated the lateral inhibition of *WOX5*, which is required to recover an activator-inhibitor motif in the root SCN GRN.

The activator-inhibitor system [81,82] is a variant of the reaction diffusion system [83]. The activator-inhibitor system consists of i) an activator that positively regulates itself and an inhibitor (in this case, *WOX5 *and *CLEX*, respectively), and ii) an inhibitor that negatively regulates the activator and has a long-range effect. It is important to note that this kind of dynamic circuit has been used to explain robustness, reappearance of patterns and self-organization in biological systems [84-88]. Several studies have suggested that such traits also characterize the root SCN. Robustness of the niche cell pattern, as discussed in the context of this paper, has indeed been observed in several mutants. For example, all *PLT *and *PIN *single mutants [11,12,46,48] have subtle effects or wild type root SCN cell structures. SCN and QC ablation experiments [62,89], on the other hand, have shown the capacity of the SCN to regenerate and suggest a self-organization capacity. Furthermore, a recent study by Sugimoto and collaborators [90] demonstrated that the structures that appear from callus regeneration experiments have cellular structures reminiscent of root tip meristems, and this fact is true if they are derived from either root or aerial organs, which strongly suggests that root tip cell structure is self-organized. The self-organized GRN proposed here constitutes a first dynamic proposal explaining the robustness and regeneration capacity observed in the *A. thaliana *root SCN.

As other authors have already pointed out, the SAM and RAM SC specification mechanisms are similar in terms of the gene families involved and the regulatory interactions observed [2,3,6]. Indeed, in both meristems, genes promoting the QC identity (*WUS *in the SAM; and *WOX5 *in the RAM) belong to the family of genes that encode for homeobox transcriptional regulators and both seem to locally self-activate and to positively regulate their inhibitors. Some lines of evidence suggest that the activator-inhibitor could account for SCN maintenance [24,25,53,91], but this experimental evidence is not sufficient to confirm this hypothesis. Interestingly, examining the experimental evidence of SC GRNs in other organisms suggests that this motif could be a generic regulatory motif for systems underlying SCN maintenance and patterning throughout multicellular eukaryotes [34,48,92,93].

### Model limitations and perspectives

All models have limitations that stem from their assumptions. For instance, the coupled GRN model suggests that the GRN underlying root SCN patterning may involve an activator-inhibitor motif. However, as we have discussed here, the SCN specification systems in the RAM of *A. thaliana *are dynamically richer than this single motif and likely incorporate several regulatory motifs, some of which could also be dynamically redundant and provide robustness to SCN pattering [60,68]. Furthermore, the module controlling the root SCN must be interconnected with other modules, not considered here, which are indispensable for its establishment and maintenance, such as those controlling hormone signal transduction pathways, the cell cycle, the recently re-described *SCHIZORIZA *gene [71,72], or other developmental modules [e.g., [55]]. Future models should prove useful for comparing the spatiotemporal dynamics of the SAM and RAM, spot their commonalities and explore what changes in gene expression patterns, gene interactions, hormone signaling, cell size and geometry or other factors could account for the different sizes, cellular structures, dynamics and morphologies of these two meristems and SCNs.

Given the fact that our models where completely discrete or were continuous approximations of the discrete version, we could not test several observed behaviors of the root and the root SCN. For example, auxin forms a gradient trough the root with the maximum concentration in the SCN, especially in the QC and columella initials, but the importance and implication of this subtle gradient was impossible to test with this model. Also, a more realistic model than this one, in which non-cell-autonomous regulation dynamically emerges rather than being pre-specified, will be helpful. Hence, future models should allow the GRN nodes' movement or other types of intercellular communication to be established and maintained dynamically rather than fixed.

Our GRN recovers the main traits of the *A. thaliana *root SCN given the available gene interaction data and some additional assumptions, thus providing the first GRN framework along with novel predictions. However, given the multiple ways in which novel interactions or nodes can be connected to the uncovered network, genomic approaches will complement the modeling approach and results put forward here and help obtain a more complete GRN underlying SCN cellular pattering in the *A. thaliana *root. It is likely that additional and redundant circuits connected to those discovered up to now and integrated in the models proposed here will yield more robust GRN models as those described for other systems [60,68].

The integration and modeling of a GRN like the one studied here will also foster work on comparative and evolutionary developmental biology. For instance, the main components of the transcriptional regulatory networks involved in SC specification in *A. thaliana *belongs to plant-specific families, but it has been found that some animal and plant developmental systems share analogous regulatory circuits [e.g., [92,93]]. Given that the SCN of all multicellular organisms share common features and that animal and plant niches share structural and dynamic traits, it will be important to uncover and dynamically characterize the GRNs involved in their maintenance in other multicellular species and examine if there are conserved or analogous regulatory motives, modules or mechanisms. These kinds of analyses would be of great value for understanding the evolution of such a system, which is key for eukaryote development, and to address questions concerning structural constraints during GRN assemblage along plant and animal evolution.

## Conclusions

We report the first GRN models capable of recovering the main traits of the *A. thaliana *root SCN cellular structure. The proposed dynamic approximation to the *A. thaliana *root SCN GRN has enabled us to detect several important gaps in the published data, some concerning the transcriptional regulators of genes considered in our GRN. These gaps involve *SCR*, *SHR*, *JKD*, *MGP *and *WOX5*, which still lack important regulators. We also detected one contradiction about *JKD *function, which we predict is not indispensable for *SCR *expression or function. Finally, we predict the existence of *WOX5 *negative regulators in the vascular initials and probably in the CEI.

Some of these predictions are amenable to experimental tests. A more robust GRN will probably imply additional components and redundant circuits. However, our models suggest that some of the key genes involved in root SCN maintenance have been discovered, but other important components remain to be found. Additional efforts on GRN simulations and genomic approaches will be fundamental for postulating more complete models for explaining the root SCN cellular patterning.

## Methods

### Boolean single cell and coupled GRNs

In the autonomous single cell and coupled GRN models, N nodes are defined and these represent the genes and molecules involved in cell patterning and maintenance of the root SCN. The state of every node can only take two possible values, *0 *(gene off) or *1 *(gene on), depending on the function:

$$x_{n}(t+\tau )=F_{n}(x_{n_{1}}(t),x_{n_{2}}(t),\ldots ,x_{n_{k}}(t))$$

For the single cell model, *x_n _*represents the state of a gene at the time (*t*+*τ*) and $\left\{x_{n_{1}}(t),x_{n_{2}}(t),\ldots ,x_{n_{k}}(t)\right\}$ represents all of the regulators of gene *x_n _*at time t. For the coupled GRN the function:

$$\begin{array}{l} x_{n}^{m}(t+\tau )=F_{n}(x_{n_{1}}^{1}(t),x_{n_{2}}^{1}(t),\\ ...,x_{n_{k}}^{1}(t),x_{n_{1}}^{2}(t),...,x_{n_{k}}^{p}(t)) \end{array}$$

defines the state of every node, where $x_{n}^{m}$ resspresents the state of a gene in a specific cell type *m *at the time (*t*+*τ*) and $\left\{x_{n_{1}}^{1}(t),x_{n_{2}}^{1}(t),...,x_{n_{k}}^{1}(t),x_{n_{1}}^{2}(t),...,x_{n_{k}}^{p}(t)\right\}$ represents all the regulators of gene $x_{n}^{m}$ at time *t *including those from other cell types capable of moving and acting non-cell autonomously. For both kinds of models, *F_n _*is a Boolean logical function based on experimental evidence. The models are deterministic and have a finite number of possible initial conditions represented by Ω (Ω = 2_*N*_). Therefore, the future states of all possible initial conditions can be determined. The models were iterated synchronously until they reached a steady state starting from all possible initial conditions.

*PLT*, *ARF *and *Aux/IAA *represent families of genes that, given their redundancy, are modeled as one node. The value of *1 *for the auxin node does not represent any concentration; rather, it represents a wild type concentration sufficient for the specific function under consideration in the model. All other nodes represent a single gene. Loss-of-function simulations were done by fixing the state of the node to *0*; for gain-of-function simulations it was set to *1*.

For the meta-GRN of the coupled GRN, we defined four domains; each one represented a SC type (Figure 4) and there was one for every attractor in the one-cell models. The logical rules enabled communication between each GRN based on experimental evidence and our activator-inhibitor prediction. In this case, we have now four coupled GRNs. Thus, when we ran the model, we expected only one global attractor.

### Continuous model

We considered a GRN with N nodes. We represented the activation level at node *k *by *X_k_*. Within a continuous scheme, the rate of change of the activation level was represented by the set of differential equations:

$$\frac{dx_{K}}{dt}=f[w_{K}(x)]-\gamma_{K}x_{K}$$

where *y_k _*is the activation decay rate, and *f*[*w_k_*(*X*_1_,...,*X_N_*)] is a logistic functional determined by the node input function *w_k_*(*X*_1_,...,*X_N_*:

$$f_{K}[w_{K}(x_{1},...,x_{N})]=\frac{1}{1+\exp[-h(w_{K}-w_{K}^{thr})]}$$

where $w_{K}^{thr}$ is the threshold activation level and h is a measure of the activation speed. Notice that in the limit $hw_{K}^{thr}\gg 1,f_{K}[w_{K}<w_{K}^{thr}]\to 0,f_{K}[w_{K}=w_{K}^{thr}]\to 1/2$, and $f_{K}[w_{K}>w_{K}^{thr}]\to 1$, so that *f_k _*behaves as a (differentiable) step-like function $f[w_{K}]≃\Theta (w_{K}-w_{K}^{thr})$. To obtain explicit solutions of the differential equation set, we assume that *y_k _*= 1, and $w_{K}^{thr}$ = 1/2. Notice, however, that the solution method applies also for arbitrary values of these parameters. The attractors of the system may be analytically derived from the condition *dx*_*K*_/*dt *= 0, which leads to a set of non-linear algebraic equations with the general form

$$x_{K}=\Theta [w_{K}(x_{1},...x_{N})]$$

which implies in turn obtaining the solutions for the cases *w*_*K *_> 1/2, *w*_*K *_= 1/2, and *w*_*K *_< 1/2. The expression for the total input at a given node, *w_i_*, representing the logical rules are given in Additional files 3 and 4. The program used for this model is freely available upon request.

## Authors' contributions

EAB conceived and coordinated the study. EAB, EA and MB designed the study. EA gathered, integrated and analyzed the experimental data and postulated the models with help from EAB and MB. EA ran the simulations for the discrete case. IV and CV established the continuous system, and IV ran its simulations. EAB, EA and MB wrote the paper. All of the authors helped interpret the results and read and validated the final version of the paper.

## Supplementary Material

Additional file 1 **This file contains the detailed topology and updating single cell GRN discrete functions**. Click here for file

Additional file 2 **This file contains additional GRN analysis under different auxin concentrations**. Click here for file

Additional file 3 **This file contains the detailed topology and updating single cell GRN continuous functions**. Click here for file

Additional file 4 **This file contains the detailed topology and updating coupled GRN discrete and continuous functions**. Click here for file
