# Supplementary material for: Single-cell and coupled GRN models of cell patterning in the Arabidopsis thaliana root stem cell niche
Source: BMC Syst Biol. 2010 Oct 5;4:134. doi: 10.1186/1752-0509-4-134 (PMC2972269; doi:10.1186/1752-0509-4-134)
Supplement: Additional file 1 — This file contains the detailed topology and updating single cell GRN discrete functions. [file 1752-0509-4-134-S1.DOC]

## Additional file 1

Logical rules of models A, A´, B and B´. The logical rules of models A and A´ and for B and B´ are presented in the same tables except for the *WOX5* node, who have different rules in models A and A´ from the once of B and B´. *CLEX* rule is exclusive of models B and B´. For models A and B the value in line 14 of *SCR* logical rule is *0*, while for A´ and B´ is *1*.

| SHR | SCR | JKD | | MGP | | | SCR |
| --- | --- | --- | --- | --- | --- | --- | --- |
| 0 | 0 | | 0 | | 0 | 0 | |
| 0 | 0 | | 0 | | 1 | 0 | |
| 0 | 0 | | 1 | | 0 | 0 | |
| 0 | 0 | | 1 | | 1 | 0 | |
| 0 | 1 | | 0 | | 0 | 0 | |
| 0 | 1 | | 0 | | 1 | 0 | |
| 0 | 1 | | 1 | | 0 | 0 | |
| 0 | 1 | | 1 | | 1 | 0 | |
| 1 | 0 | | 0 | | 0 | 0 | |
| 1 | 0 | | 0 | | 1 | 0 | |
| 1 | 0 | | 1 | | 0 | 0 | |
| 1 | 0 | | 1 | | 1 | 0 | |
| 1 | 1 | | 0 | | 0 | 1 | |
| 1 | 1 | | 0 | | 1 | 1/0 | |
| 1 | 1 | | 1 | | 0 | 1 | |
| 1 | 1 | | 1 | | 1 | 1 | |

| ARF | PLT |
| --- | --- |
| 0 | 0 |
| 1 | 1 |

| Aux/IAA | ARF |
| --- | --- |
| 0 | 1 |
| 1 | 0 |

| Auxin | Aux/IAA |
| --- | --- |
| 0 | 1 |
| 1 | 0 |

| Auxin | Auxin |
| --- | --- |
| 0 | 1 |
| 1 | 1 |

| SHR | SHR |
| --- | --- |
| 0 | 0 |
| 1 | 1 |

| SHR | SCR | JKD |
| --- | --- | --- |
| 0 | 0 | 0 |
| 0 | 1 | 0 |
| 1 | 0 | 0 |
| 1 | 1 | 1 |

| SHR | SCR | WOX5 | MGP |
| --- | --- | --- | --- |
| 0 | 0 | 0 | 0 |
| 0 | 0 | 1 | 0 |
| 0 | 0 | 0 | 0 |
| 0 | 0 | 1 | 0 |
| 1 | 1 | 0 | 0 |
| 1 | 1 | 1 | 0 |
| 1 | 1 | 0 | 1 |
| 1 | 1 | 1 | 0 |

## CLEX rule (only for models B and B`)

| SHR | CLEX | CLEX |
| --- | --- | --- |
| 0 | 0 | 0 |
| 0 | 1 | 0 |
| 1 | 0 | 0 |
| 1 | 1 | 1 |

## WOX5 rule for models A and A`

| ARF | SHR | SCR | MGP | WOX5 | WOX5 |
| --- | --- | --- | --- | --- | --- |
| 0 | 0 | 0 | 0 | 0 | 0 |
| 0 | 0 | 0 | 0 | 1 | 0 |
| 0 | 0 | 0 | 1 | 0 | 0 |
| 0 | 0 | 0 | 1 | 1 | 0 |
| 0 | 0 | 1 | 0 | 0 | 0 |
| 0 | 0 | 1 | 0 | 1 | 0 |
| 0 | 0 | 1 | 1 | 0 | 0 |
| 0 | 0 | 1 | 1 | 1 | 0 |
| 0 | 1 | 0 | 0 | 0 | 0 |
| 0 | 1 | 0 | 0 | 1 | 0 |
| 0 | 1 | 0 | 1 | 0 | 0 |
| 0 | 1 | 0 | 1 | 1 | 0 |
| 0 | 1 | 1 | 0 | 0 | 0 |
| 0 | 1 | 1 | 0 | 1 | 0 |
| 0 | 1 | 1 | 1 | 0 | 0 |
| 0 | 1 | 1 | 1 | 1 | 0 |
| 1 | 0 | 0 | 0 | 0 | 0 |
| 1 | 0 | 0 | 0 | 1 | 0 |
| 1 | 0 | 0 | 1 | 0 | 0 |
| 1 | 0 | 0 | 1 | 1 | 0 |
| 1 | 0 | 1 | 0 | 0 | 0 |
| 1 | 0 | 1 | 0 | 1 | 0 |
| 1 | 0 | 1 | 1 | 0 | 0 |
| 1 | 0 | 1 | 1 | 1 | 0 |
| 1 | 1 | 0 | 0 | 0 | 0 |
| 1 | 1 | 0 | 0 | 1 | 0 |
| 1 | 1 | 0 | 1 | 0 | 0 |
| 1 | 1 | 0 | 1 | 1 | 0 |
| 1 | 1 | 1 | 0 | 0 | 1 |
| 1 | 1 | 1 | 0 | 1 | 1 |
| 1 | 1 | 1 | 1 | 0 | 0 |
| 1 | 1 | 1 | 1 | 1 | 1 |

## WOX5 rule for models B and B´

| ARF | SHR | SCR | CLEX | WOX5 |
| --- | --- | --- | --- | --- |
| 0 | 0 | 0 | 0 | 0 |
| 0 | 0 | 0 | 1 | 0 |
| 0 | 0 | 1 | 0 | 0 |
| 0 | 0 | 1 | 1 | 0 |
| 0 | 1 | 0 | 0 | 0 |
| 0 | 1 | 0 | 1 | 0 |
| 0 | 1 | 1 | 0 | 0 |
| 0 | 1 | 1 | 1 | 0 |
| 1 | 0 | 0 | 0 | 0 |
| 1 | 0 | 0 | 1 | 0 |
| 1 | 0 | 1 | 0 | 0 |
| 1 | 0 | 1 | 1 | 0 |
| 1 | 1 | 0 | 0 | 0 |
| 1 | 1 | 0 | 1 | 0 |
| 1 | 1 | 1 | 0 | 1 |
| 1 | 1 | 1 | 1 | 0 |
