# Supplementary material for: Single-cell and coupled GRN models of cell patterning in the Arabidopsis thaliana root stem cell niche
Source: BMC Syst Biol. 2010 Oct 5;4:134. doi: 10.1186/1752-0509-4-134 (PMC2972269; doi:10.1186/1752-0509-4-134)
Supplement: Additional file 2 — This file contains additional GRN analysis under different auxin concentrations. [file 1752-0509-4-134-S2.DOC]

**Additional file 2 - Auxin control of apical-basal behavior in the Arabidopsis root meristem**

We also used models A, A`, B and B` to test the effects of varying auxin concentrations. Even though it is well known that auxins have a gradient-type expression pattern along the apical-basal axis of the root, we assumed that changing the value of the node between *0* and *1* in our model would represent different auxin availability. By modeling auxin availability in this way, we could explore alterations in the dynamics of the GRN involved in cell fate determination within the root SCN along the apical-basal axis of the entire RAM. Given the genes currently incorporated into the proposed SCN GRN module, we expected seven different attractors or stable configurations: the four found and described for the SCN plus those corresponding to vascular and endodermis cells as well as one corresponding to cortex-epidermis-lateral-root cap-columella cells. In all of the simulations of the SCN pattern that we present in the main text, auxin was fixed to *1* because the SCN has the highest concentration of this hormone in the root [1]. However, when we allowed the node corresponding to auxin to vary between *0* and *1*, we recovered six of the seven expected attractors. One configuration did not correspond to any known cellular type of the root as it presented endodermis characteristics that were not in agreement with experimental data because *MGP* stayed “ON”. Our GRN does not have a negative regulatory control over *MGP* in the transit towards the meristematic or amplifying zone, and its expression depends only on *SCR* and *SHR*, which are both expressed in the endodermis. Consequently, we tested the effect of including additional hypothetical genes and found two experimentally testable forms that recovered the observed gene expression configurations. The first and most parsimonious case requires the introduction of a hypothetical gene that represses the activation of *MGP*, and it could be any endodermal identity gene. The second case hypothesizes *LHP1* as a repressor of *MGP*, as suggested in Cui and Benfey [2]. We believe that MGP expression could depend on another gene expressed specifically in the SCN, but we were unable to test this because we did not find a way to express a hypothetical gene in the SCN exclusively and, more specifically, only in the QC and CEI.

Next, we performed the perturbation analyses in these two variants of the model in all four versions and found that only 62.5%, 61.7% and 54.3% were neutral lines in the logical rule in the version of the models for the first hypothesis for model A, A` and B (both versions), respectively, and 62.7%, 62.5% 54.3 % and 55.1% in the version of the models for the second hypothesis for models A, A`, B and B`, respectively. We then introduced *PINX* under the control of the *PLT* genes and found that even when *PLT* and *ARF* genes are necessary for PIN1 expression, there must be another positive regulator for the *PIN* genes in general, as already suggested by the experimental data.

**Additional references**

## 1. Sabatini S, Beis D, Wolkenfelt H, Murfett J, Guilfoyle T, Malamy J, Benfey P, Leyser O, Bechtold N, Weisbeek P, Scheres B: An auxin-dependent distal organizer of pattern and polarity in the Arabidopsis root. Cell 1999, 99:463-72.

## 2. **Cui H, Benfey PN:** Interplay between SCARECROW, GA and LIKE HETEROCHROMATIN PROTEIN 1 in ground tissue patterning in the Arabidopsis root**. Plant J 2009,** 58**:1016-27**
