# Supplementary material for: Single-cell and coupled GRN models of cell patterning in the Arabidopsis thaliana root stem cell niche
Source: BMC Syst Biol. 2010 Oct 5;4:134. doi: 10.1186/1752-0509-4-134 (PMC2972269; doi:10.1186/1752-0509-4-134)
Supplement: Additional file 3 — This file contains the detailed topology and updating single cell GRN continuous functions. [file 1752-0509-4-134-S3.DOC]

## Additional file 3

## Estimation of the *w* parameter from logical rules. This parameter characterizes the equation of each gene in the continuous GRN model.

## 
