# Supplementary material for: Single-cell and coupled GRN models of cell patterning in the Arabidopsis thaliana root stem cell niche
Source: BMC Syst Biol. 2010 Oct 5;4:134. doi: 10.1186/1752-0509-4-134 (PMC2972269; doi:10.1186/1752-0509-4-134)
Supplement: Additional file 4 — This file contains the detailed topology and updating coupled GRN discrete and continuous functions. [file 1752-0509-4-134-S4.DOC]

## Additional file 4.

## Logical rules and estimation of the *w* parameter from logical rules used in the coupled GRN model.

PLT = ARF in all cell types

Auxin = 1 in Vascular initials and CEI

Auxin = Vascular initials Auxin OR CEI Auxin in QC

Auxin = QC Auxin in CEpI

Aux/IAA = NO Auxin AND NO WOX5 in all cell types

ARF = NO Aux/IAA in all cell types

SHR = 1 in vascular initials

SHR = Vascular initials SHR in QC and CEI

SHR = (Vascular initials SHR AND NO vascular initials SCR) OR (QC SHR and NO QC SCR) in CEpI

SCR = 0 in vascular initials

SCR = SHR in QC and CEI

SCR = SHR AND SCR in CEpI

MGP = (SHR AND SCR) AND NO WOX5 in all cell types

JKD = SHR AND SCR in all cell types

WOX5 = ((ARF AND SHR) AND SCR) AND NO CLEX) in all cell types

CLEX = QC WOX5 AND CLEX in vascular initials, CEI and CEpI

CLEX = 0 in QC

$$\begin{matrix} w_{QAUX}=\max(VAUX,CAUX) \\ w_{QIAA}=\min(1-QAUX,1-WWOX5) \\ w_{QARF}=1-QIAA \\ w_{QPLT}=QARF \\ w_{QSHR}=VSHRT \\ w_{QSCR}=\min(QSHR,QSCR) \\ w_{QJKD}=\min(QSHR,QSCR) \\ w_{QMGP}=\min[\min(QSHR,QSCR),1-QWOX5)] \\ w_{QWOX5}=\min[\min(QARF,QSHR,QSCR),1-QCLEX] \\ w_{QCLEX}=0 \end{matrix}$$

$$\begin{matrix} w_{VAUX}=1 \\ w_{VIAA}=\min(1-VAUX,1-VWOX5) \\ w_{VARF}=1-VIAA \\ w_{VPLT}=VARF \\ w_{VSHR}=1 \\ w_{VSCR}=0 \\ w_{VJKD}=\min(VSHR,VSCR) \\ w_{VMGP}=\min[\min(VSHR,VSCR),1-VWOX5)] \\ w_{VWOX5}=\min[\min(VARF,VSHR,VSCR),1-VCLEX] \\ w_{VCLEX}=QWOX5 \end{matrix}$$

$$\begin{matrix} w_{CAUX}=1 \\ w_{CIAA}=\min(1-CAUX,1-CWOX5) \\ w_{CARF}=1-CIAA \\ w_{CPLT}=CARF \\ w_{CSHR}=VSHR \\ w_{CSCR}=\min(CSHR,CSCR) \\ w_{CJKD}=\min(CSHR,CSCR) \\ w_{CMGP}=\min[\min(VSHR,VSCR),1-VWOX5)] \\ w_{CWOX5}=\min[\min(CARF,CSHR,CSCR),1-CCLEX] \\ w_{CCLEX}=QWOX5 \end{matrix}$$

$$\begin{matrix} w_{MAUX}=QAUX \\ w_{MIAA}=\min(1-MAUX,1-MWOX5) \\ w_{MARF}=1-MIAA \\ w_{MPLT}=MARF \\ w_{MSHR}=\min[\min(QSHR,1-QSCR),\min(CSHR,CSCR)] \\ w_{MSCR}=\min(MSHR,MSCR) \\ w_{MJKD}=\min(MSHR,MSCR) \\ w_{MMGP}=\min[\min(MSHR,MSCR),1-MWOX5)] \\ w_{MWOX5}=\min[\min(MARF,MSHR,MSCR),1-MCLEX] \\ w_{MCLEX}=QWOX5 \end{matrix}$$
